# Randomized *in vivo* trial evaluating plaque inhibition benefits of an advanced stannous-containing sodium fluoride dentifrice used in conjunction with power brush technology

**DOI:** 10.1111/idh.12040

**Published:** 2013-07-12

**Authors:** PG Bellamy, A Boulding, S Farmer, TN Day, ML Barker, R Harris, AJ Mussett

**Affiliations:** Procter & Gamble, Greater London Innovation CenterReading, UK; Procter & Gamble, Greater London Innovation CentreEgham, UK; Procter & Gamble, Mason Business CenterMason, OH, USA; Colour Bright, Ltd.Egham, UK

**Keywords:** digital plaque imaging analysis, electric toothbrush, plaque imaging, plaque inhibition, power toothbrush, stannous chloride, stannous fluoride

## Abstract

**Objective:**

To compare the plaque inhibition efficacy of a novel stannous-containing sodium fluoride test dentifrice to a standard anticavity negative control dentifrice, when both were used in conjunction with an advanced oscillating–rotating (O/R) power toothbrush.

**Methods:**

This was a randomized, two-treatment, three-period, double-blind crossover study conducted in a population using an O/R power brush. Subjects brushed twice per day with their assigned dentifrice during the three-treatment periods, each lasting for 17 consecutive days. Each period was separated by a 4-day washout period during which subjects continued to use their O/R power toothbrush. Plaque levels were assessed and averaged amongst three assessments taken on days 15, 16 and 17 at the end of each treatment period using digital plaque imaging analysis. Assessments were carried out on the facial anterior tooth surfaces in the morning before brushing (A.M. prebrush) following whole-mouth brushing (30 s per quadrant) with the assigned dentifrice (A.M. post-brush) and in the afternoon (P.M.).

**Results:**

Twenty-seven subjects were randomized and completed the study. During the 17-day usage period, the stannous-containing test NaF dentifrice demonstrated a statistically significant lower mean plaque area versus the negative control dentifrice at each assessment timepoint; overnight A.M. prebrush was 33.8% lower (*P* < 0.0001), A.M. post-brush was 21.8% lower (*P* < 0.01), and P.M. was 29.2% lower (*P* < 0.0001).

**Conclusion:**

A population of O/R power toothbrush users had significantly less plaque coverage for all three measurements when using a stannous-containing NaF dentifrice than when using a negative control (fluoride) dentifrice.

## Introduction

The results of the numerous comparative clinical studies of oral hygiene products conducted to date give valuable guidance to manufacturers seeking to optimize product effectiveness. Crucially, these studies also provide essential information to dental professionals and patients who are faced with a vast array of marketed products and wish to differentiate between them. Given the fundamental importance of effective plaque control for achieving good oral health and the prevention of periodontal disease 1–5, it is not surprising that the development of technologies to optimize daily plaque removal continues to be a common focus for manufacturers of oral hygiene products.

The introduction of the power toothbrush was a notable advance as it offered a technology with the potential for achieving highly effective plaque removal while aiming to make mechanical brushing a more positive experience. Since power brushes were first introduced, a variety of designs have become available with different bristle and brush head motion (e.g. side-to-side or oscillating–rotating) 6,7. Many clinical studies have assessed the comparative clinical effectiveness of the various models, and a well-documented comprehensive review of the results of comparative clinical studies has shown plaque control advantages for power brushes over manual toothbrushes, but only those power toothbrushes that have an oscillating–rotating action have shown consistent short- and long-term benefits for plaque removal and gingivitis reduction 8–12.

Advances in toothbrush technology are associated with more effective plaque removal, but excessive plaque regrowth can also be a problem for individuals 13. Therefore, there is a need for products that not only help users to achieve optimal plaque removal, but also ensure that plaque levels remain controlled overnight and throughout the day, thereby reducing the risk of oral hygiene becoming suboptimal. The choice of dentifrice has been shown to have a significant effect on the inhibition of plaque regrowth in a study with manual toothbrushes and may also play an important role in optimizing the level of plaque control achieved with power brushing 14–18.

Dentifrices that can offer multiple benefits in one product are becoming increasingly common, and a novel stannous-containing NaF dentifrice (marketed as blend-a-med Pro-Expert in parts of Europe and Crest Pro-Health in China) has recently been marketed as one that provides anticaries as well as antimicrobial properties, along with other benefits including desensitizing and breath malodour advantages 19–23. In the present study, the plaque inhibition effectiveness of this dentifrice was compared with that of a standard anticavity fluoride dentifrice (Colgate Cavity Protection; Colgate-Palmolive Co., Guilford, UK) when used with a leading power toothbrush (Oral-B Triumph with SmartGuide; Procter & Gamble Company, Cincinnati, OH, USA) in a randomized, double-blind, crossover study with three 17-day treatment periods. Plaque levels were assessed using white-light digital plaque imaging analysis (DPIA) as this method is established as a valid sensitive, objective method for measuring plaque in clinical studies to differentiate between dentifrices 14–18,24.

## Materials and methods

### Treatment products

The test dentifrice contained sodium fluoride (NaF, 1450 ppm F) as the active ingredient and stannous chloride as a key excipient (blend-a-med Pro-Expert dentifrice; Procter & Gamble, Gross Gerau, Germany). These two elements combine synergistically during the act of toothbrushing to generate a stannous fluoride complex. The comparison negative control dentifrice contained a dual fluoride source, with 1000 ppm fluoride provided by sodium monofluorophosphate and a further 450 ppm fluoride provided by NaF (Colgate Cavity Protection, Colgate-Palmolive Co.).

### Study design and procedures

**Figure 1:**
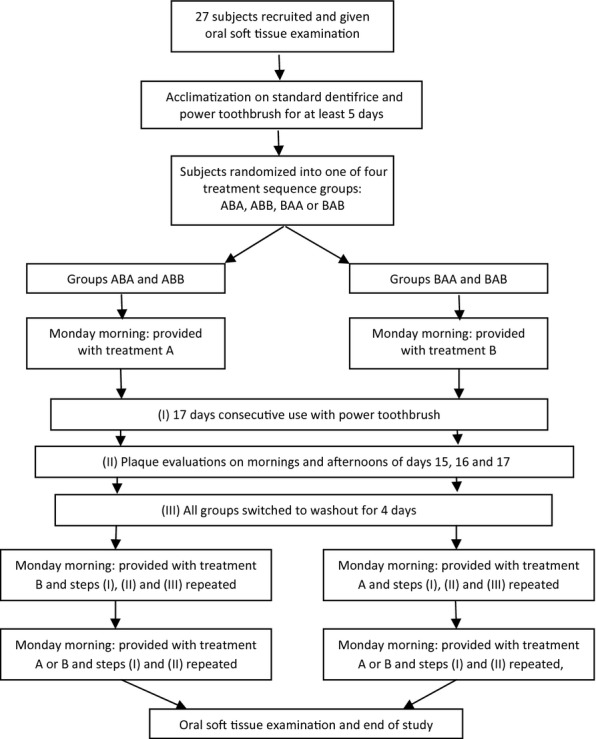
Study design.

Subjects gave their signed informed consent before the start of any study procedures, and the use of DPIA methodology in dentifrice research had been approved by the Institutional Ethics Review Committee. The subjects for this study were 27 employees at Procter & Gamble, Egham, UK, and all had previously participated in plaque trials using DPIA methodology. The study was conducted over a period of 10 weeks and had a double-blind, two-treatment, three-period, randomized crossover design similar to that described by Bellamy *et al*. 17 (Fig.1). Digital plaque imaging analysis is an objective method for assessing plaque. Although an employee population was used, objective assessment and the double-blind randomized nature of the design greatly reduce the chance of a population bias affecting the results.

An acclimatization period of at least 5 days was followed by three 17-day treatment periods that were separated by washout periods of 4 days. During washout periods, all subjects used a 1450 ppm NaF toothpaste (Crest® Decay Prevention; Procter & Gamble) and continued using the provided Oral-B power toothbrush. Plaque evaluations using DPIA methodology were carried out before and after brushing (A.M. prebrush and A.M. post-brush) and in the afternoon (P.M.) on days 15, 16 and 17 of each period to give subjects time to acclimatize to their treatment products.

Following their informed consent, subjects received an oral soft tissue examination and were assessed according to a number of study inclusion/exclusion criteria. To be included in the study, subjects were required to demonstrate plaque accumulation overnight as assessed with white-light DPIA when using a standard fluoride toothpaste. Subjects were also required to be in good general health, to agree to use the treatment products as directed and brush at a consistent timepoint every evening and to refrain from eating or drinking (except water) on DPIA assessment mornings or within 30 min of an afternoon DPIA assessment. The use of oral hygiene products (including use of chewing gum and dental floss) was not allowed, but floss users could continue to floss their back teeth only (i.e. those not imaged) providing they did so consistently throughout the study. Subjects with poor dental health (e.g. rampant caries, severe gingivitis) or known dye allergies (especially fluorescein) and those who were pregnant or nursing were excluded, as were subjects who used medications (e.g. antibiotics, antimicrobial mouthwash, medicated lozenges) 2 weeks prior to the study or at any stage during the study. Also excluded were subjects with dental conditions, such as orthodontic appliances, that would interfere with the study and those with colour-matched restorations present on more than three teeth in the facial anterior arch.

Subjects used a standard NaF dentifrice (Crest® Decay Prevention; Procter & Gamble) and power brush (Oral-B® Triumph™ 5000 with EB17 brush head plus SmartGuide™; Procter & Gamble Company) during the initial 5-day acclimatization period and during each of the 2, 4-day washout periods between the treatment periods. At the start of the acclimatization period, subjects were instructed to brush twice a day (i.e. morning and just before going to bed in the evening) and to brush the whole of the mouth quadrant by quadrant (30 s per quadrant) following the power brush instructions using a full brush head of toothpaste.

On the first day (a Monday) of the first 17-day treatment period (period 1), subjects were randomized into one of four treatment sequence groups (ABB, BAA, ABA or BAB, where A and B represent the two-treatment dentifrice products). The stannous-containing NaF test dentifrice and the negative control comparison dentifrice (A and B) were supplied in white unbranded packaging to ensure treatment was fully blinded. Subjects were instructed to use their assigned treatment product starting that evening, and the treatment brushing instructions were the same as for the acclimatization period. Days 15, 16 and 17 (Tuesday to Thursday) of each treatment period were assessment days for the evaluation of plaque by DPIA, and subjects were instructed not to brush, eat or drink (except water) on assessment mornings. On these mornings and without brushing their teeth, subjects attended for clinic imaging appointments. Subjects disclosed plaque by rinsing the mouth for 10 s with 25 ml of phosphate buffer (pH 6.0). This was followed by a 1-minute rinse with 5 ml of disclosing dye (fluorescein diacetate solution, made fresh daily) and in turn was followed by three further 10-second rinses with 25 ml of phosphate buffer. Digital plaque imaging analysis was used between 30 s and 1 min after the final buffer rinse to capture the A.M. prebrushing disclosed plaque on the facial anterior surfaces of the 12 anterior teeth (canine to canine: six mandibular and six maxillary). Subjects then used a full brush head of their assigned treatment product (dentifrice A or B) and power brush (supplied in the imaging area, so there was no need for subjects to bring their products from home) to brush the whole of their mouth quadrant by quadrant (30 s per quadrant) following the power brush instructions. Plaque was then redisclosed with dye solution as before, and the A.M. post-brushing image was captured by DPIA. Subjects returned to the clinic in the afternoon (between 1.30 and 3.30 P.M.) when they disclosed their plaque as before and had a further DPIA assessment (P.M.). Subjects were required not to eat or drink for 30 min following a DPIA assessment or for 30 min before the afternoon DPIA assessment so as not to interfere with the assessment process, but otherwise were free to eat and drink as normal.

Subjects returned to using the standard NaF dentifrice and power brush during the 4-day washout phase of the study between the evening of day 17 (period 1) and the start of the second 17-day treatment period the following Monday (period 2) and similarly for the second 4-day washout period (between periods 2 and 3). The methods for each of the 3-treatment crossover periods were the same except that the subject used the assigned treatment product according to the randomization schedule. Period 3 was followed by a final soft tissue examination.

### Image analysis

The DPIA procedure has been described by Bellamy *et al*. 14–17 and targets the facial anterior surfaces of the 12 clearly visible teeth (six mandibular and six maxillary; canine to canine). Every pixel in the predefined region of interest was assigned to one of four classes (tooth, gums, plaque or background) using a computerized analysis routine, and the results were checked for consistency and accuracy by an experienced image analysis operator blinded to the assigned treatment. The following equation was used to calculate the percentage of the tooth area covered with plaque:

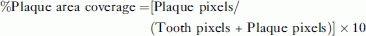


### Statistical methods

Previous studies using this design have demonstrated that a study base size greater than 25 is sufficient to consistently show differences between products similar to those being tested in this trial 14–18.

The percentage plaque area coverage measurements from each of 3 days were averaged separately for each subject, period and timepoint (A.M. prebrush, A.M. post-brush and P.M.). For each timepoint, analysis of variance for the crossover design (general linear mixed model) was used to compare the percentage plaque area coverage between treatments using period and treatment dentifrice as fixed effects and subject as a random effect. The carryover effect was tested for each timepoint and was found to be statistically significant (*P* < 0.005) and was included as a fixed effect in each statistical model. All statistical comparisons were two-sided using a 0.05 significance level.

## Results

In total, 27 subjects were enrolled in the study, and all subjects completed all measurements with the exception of six subjects that each missed all three timepoints from one study period (one subject from period 1, three subjects from period 2 and two subjects from period 3). Despite the missing observations, the number of measurements for each treatment dentifrice was still approximately equal. In addition for periods 2 and 3, the number of observations where each treatment dentifrice was used in the prior period was approximately equal. All images were considered to be of sufficient quality to be included in analysis, and no images needed to be excluded during analysis due to poor classification by the computer algorithm. No adverse events were recorded by the investigator, and no product use discomfort was reported by the subjects. Subjects ranged in age from 25 to 57 years with a mean of 35.3 years; 63% of the subjects were female (Table1).

**Table 1:** Subject demographic characteristics

Gender: *n* (%)		Age: years	
Male	Female	Mean (SD)	Min–max
10 (37)	17 (63)	35.3 (7.82)	25–57

SD, standard deviation.

The general linear mixed model adjusted for period and carryover effects to compare treatment dentifrices during the 17-day usage period with an advanced power toothbrush. In this research for each timepoint, a statistically significant (*P* < 0.005) carryover effect determined that the stannous-containing NaF dentifrice had less plaque in the period that followed its use relative to the negative control dentifrice. For each timepoint, a statistically significant period effect (overall *P*-value <0.05) was observed in the mean plaque level and tended to increase over the course of the study (Table2). Even though the carryover and period effects were statistically significant, the general linear mixed model adjusted for these fixed effects and estimated the appropriate comparison between the treatment dentifrices.

**Table 2:** Period comparison of plaque area (%) during 17 days of product use with a power toothbrush

Timepoint and treatment	Estimated mean (SE)	*P*-value versus period 2*	*P*-value versus period 3*
A.M. prebrush (carryover effect *P*-value = 0.0039)			
Period 1	7.42 (1.16)	0.0021	0.0003
Period 2	10.21 (1.18)	0.5764	
Period 3	10.70 (1.17)		
A.M. post-brush (carryover effect *P*-value = 0.0013)			
Period 1	2.98 (0.64)	0.0050	0.0016
Period 2	3.97 (0.65)	0.7279	
Period 3	4.09 (0.65)		
P.M. (carryover effect *P*-value = 0.0046)			
Period 1	5.66 (0.80)	0.1369	0.0128
Period 2	6.44 (0.81)	0.3090	
Period 3	6.98 (0.81)		

SE, standard error.

* Two-sided *P*-value; analysis of variance for crossover design.

During the 17-day usage period with the advanced power toothbrush, subjects demonstrated significantly lower plaque area coverage while using the stannous-containing NaF than while using the negative control dentifrice. This was true for all three timepoints: A.M. prebrushing, A.M. post-brushing and P.M. Table3 shows the mean plaque coverage for each timepoint and the statistical treatment comparison between the products.

**Table 3:** Treatment comparison of plaque area (%) during 17 days of product use with a power toothbrush

Timepoint and treatment	Estimated mean (SE)	Relative% reduction	Treatment comparison *P*-value*
A.M. prebrush			
Stannous-containing NaF	7.53 (1.13)	33.76	<0.0001
Negative control	11.37 (1.13)		
A.M. post-brush			
Stannous-containing NaF	3.23 (0.64)	21.79	0.0057
Negative control	4.13 (0.64)		
P.M.			
Stannous-containing NaF	5.27 (0.79)	29.21	<0.0001
Negative control	7.45 (0.78)		

SE, standard error.

Two-sided *P*-value; analysis of variance for crossover design.

Overnight A.M. prebrushing plaque growth when subjects were using the stannous-containing NaF dentifrice was significantly (*P* < 0.0001) lower on average by 33.8% than when subjects used the negative control dentifrice with estimated means (SE) of 7.53 (1.13) and 11.37 (1.13), respectively. Immediately after brushing with the advanced power toothbrush, the stannous-containing NaF dentifrice demonstrated significantly (*P* < 0.0057) less plaque coverage by 21.8% relative to the negative control dentifrice with estimated means (SE) of 3.23 (0.64) and 4.13 (0.64), respectively. After a period of daytime plaque regrowth, the stannous-containing NaF dentifrice had significantly (*P* < 0.0001) less plaque coverage on average by 29.2% versus the negative control dentifrice with estimated means (SE) of 5.27 (0.79) and 7.45 (0.78), respectively.

## Discussion

Use of power (or electric) toothbrushes is becoming increasingly common. In recent years, a number of countries have shown rapid increases in power brush usage. For example, in the UK, over 25% of households now contain a power toothbrush, while the equivalent figure in Germany is over 33% (manufacturer data). Research data on O/R power toothbrushes have been independently reviewed by the Cochrane Collaboration 8–10, who found evidence supporting the benefits of this type of power brushing technology for the reduction in plaque and gingivitis.

This poses an interesting question. Now that a substantial proportion of people in some countries are regularly using power toothbrushes for their daily oral hygiene, do dentifrices containing antimicrobials provide any additional benefit? Most research investigating the efficacy of antimicrobial toothpastes understandably has evaluated the dentifrice with a standard flat profile manual toothbrush (e.g. ADA reference brush or Oral-B Indicator) to assess the efficacy of the dentifrice alone using conditions that mimicked typical use conditions.

The study described in this article investigated this question by comparing a previously proven antimicrobial toothpaste to a standard fluoride toothpaste in a population who used O/R power toothbrushes throughout the duration of the study, including during the acclimatization phase and the between treatment washout periods. This therefore could be considered analogous to the rapidly growing population of regular power brush users.

The use of stannous fluoride and stannous-containing NaF toothpaste formulations is becoming more widespread. Both types of dentifrice formulations have been proved to be effective at inhibiting plaque growth 14–18,20. In particular, studies have been conducted using a very similar design and method of plaque evaluation employed in this study. Two examples are Bellamy *et al*. 15 and Bellamy *et al*. 16. In the former, it was shown that the plaque prevention properties of a stannous fluoride (with sodium hexametaphosphate) toothpaste were significantly greater than a NaF/potassium nitrate control dentifrice. In the latter study, a stannous-containing NaF toothpaste was also shown to be superior to a NaF/potassium nitrate control dentifrice at preventing plaque regrowth overnight and during the day. In both these studies, the study population used a flat profile manual toothbrush (Oral-B Indicator). Results showed that test dentifrices containing stannous with NaF or stannous fluoride showed the ability to slow plaque regrowth compared with the control product. Prebrushing AM plaque was 23.3 and 26.0% lower in the two trials, respectively.

In this study, the population used a premium O/R power brush (Oral-B Triumph; Procter & Gamble Company) as per the manufacturers’ recommended instructions (2-min brushing, 30s per quadrant), with a wireless ‘SmartGuide’ to help maintain compliance and technique. Using a double-blind, randomized, crossover design, subjects used both dentifrices for 17 days either once or twice, depending on their treatment sequence. Plaque evaluations were conducted prebrushing in the early morning to assess overnight plaque growth and during the afternoon to assess daytime plaque growth. Additionally, after brushing (morning only), evaluations were made. At all three measured timepoints, subjects using the stannous-containing NaF dentifrice had significantly less plaque than when using the negative control toothpaste (A.M. prebrush 33.8% lower, *P* < 0.0001; P.M. 29.2% lower, *P* < 0.0001; A.M. post-brush 21.8% lower, *P* < 0.0057, respectively). Although a period effect was present, this was accounted for in the statistical model, and the dentifrice treatment effect was still highly significant. The results of the present study show that in a population using an O/R power brush, the benefits of stannous-containing toothpastes can still be objectively demonstrated. These results are comparable to the Bellamy 2009 and 2010 studies (both conducted with manual brushes) and strongly suggest that the antimicrobial benefits seen from stannous with NaF and SnF_2_ toothpastes amongst manual brush-using populations remain present in populations using O/R power brush technology.

It is encouraging that advances are being made not only in toothbrush and dentifrice technology, but also in the means by which plaque levels can be assessed in clinical studies. DPIA, with either UV or white-light illumination, is proving to be a convenient and reliable means of measuring plaque that is objective and sensitive enough to reveal significant differences in effectiveness between dentifrices 14–18,24. While this study was conducted with an experienced panel, similar results would be expected using a randomly selected panel given the objective nature of the DPIA methodology. Identifying these differences is important for ensuring that professionals and patients can make choices between dental hygiene products based on evidence of clinically demonstrated benefits. The current generation of oscillating–rotating power toothbrushes used in conjunction with the appropriate dentifrice for plaque prevention together represents a significant means of plaque control and may provide a preferred solution for an effective daily oral health care regimen. Ultimately, professionals and patients will choose the product most suited to their individual circumstances, but comparative clinical studies are the means by which a seemingly overwhelming variety of available products can be differentiated in terms of clinically proven effectiveness.

## Conclusion

This study showed that in a population brushing with an advanced oscillating–rotating power toothbrush, there was significantly less plaque coverage when used in combination with a stannous-containing NaF dentifrice than when used with a standard negative control fluoride toothpaste. Specifically, there was less plaque regrowth overnight and during the day, and there was also less plaque coverage immediately after brushing. The plaque inhibition effects of the stannous-containing NaF dentifrice found in this study showed that the dentifrice could enhance the previously proven plaque control benefits of leading power toothbrush technology.
